# Introjective Individuals Tend Toward Anhedonia: Self-Report and Experimental Evidence

**DOI:** 10.3389/fpsyt.2018.00298

**Published:** 2018-07-05

**Authors:** Jaime R. Silva, Anastassia Vivanco-Carlevari, Claudio Martínez, Mariane Krause

**Affiliations:** ^1^Centro de Apego y Regulación Emocional, Universidad del Desarrollo, Santiago, Chile; ^2^Clínica Alemana de Santiago, Santiago, Chile; ^3^Facultad de Psicología, Universidad Diego Portales, Santiago, Chile; ^4^Escuela de Psicología, Pontificia Universidad Católica de Chile, Santiago, Chile

**Keywords:** polarities of experience, depression, anhedonia, reward sensitivity, introjective character

## Abstract

A broad line of research has conceptualized personality based on the interaction of two aspects: interpersonal relatedness and self-definition. This theoretical corpus understands these functions as two poles according to the patterns of interaction and relevance in personality. Additionally, the exacerbation of one of these poles generates a psychopathological model that identifies three types of depressive experience: anaclitic, introjective, or mixed pattern. Understanding the lack of interest as a key symptom of depression, this experiment evaluates a relation for anhedonia and the polarities model configuration using an empirical and experimental protocol. We tested 177 individuals using the Beck Depression Inventory (BDI) subscale for anhedonia and a visual discrimination task with a specific reward system, which was implemented to study reinforcement sensitivity. Participants were classified into four groups by the polarities of experience model. The subscale's results showed that individuals with an introjective character exhibited an enhanced anhedonic symptomatology but no co-occurrence of this evidence on the experimental protocol. These results empirically support the two polarities of the depressive personality model and raise new questions regarding how to experimentally test this relation.

## Introduction

Blatt's (1) Polarities of Experience (POE) model has been widely recognized as a clinical and theoretical approach based on how individuals deal with interpersonal relationships and build a sense of identity. This conceptual scheme has taken these two cornerstones as poles from which individuals organize their experience. These two poles are branched in three different characters: anaclitic, introjective, and mixed anaclitic/introjective (2, 3). The POE model has developed substantial literature regarding how these three characters construct a particular depressive configuration depending on whether such experience is focused on interpersonal relatedness or self-definition (1, 2, 4, 5). Importantly, in POE literature, anaclitic depression manifests through an excessive emphasis on interpersonal relatedness, leading to feelings of loneliness, low self-criticism, helplessness, and weakness (6). In contrast, introjective depression is determined by the pole of self-definition, generating high self-criticism, anhedonia, guilt, and feelings of inferiority/failure (7). Mixed anaclitic/introjective depression (mixed AI) involves combined experiences of both polarities. Several studies (7–10) have revealed substantial differences in specific cognitive and affective parameters in these forms of depression, emphasizing the clinical utility of this classification (4, 11).

Furthermore, there is a significant body of evidence about the lack of interest in positive experiences (anhedonia) in depression, which is an essential component of many diagnoses (12, 13). It is important to note that anhedonia should not be conceptualized only as a loss of pleasure but also as a lack of reactivity to pleasurable stimuli or response to reward (13). Thus, studies investigating depression from the POE approach have orthogonally associated anhedonia with introjective patterns (7, 11).

Considering the important clinical and theoretical contributions of the POE model, little has been studied experimentally (14). In this line, this study aimed to examine how the different POE characters react to reinforcement—in particular, whether introjective character is associated with diminished motivation for rewards (anhedonia). To this end, after we applied the Depressive Experiences Questionnaire (DEQ, (15)) to set the participants according to the POE characters, we investigated their anhedonic pattern in two ways: first, according to an anhedonia subscale from Beck's Depression Inventory (BDI, (16)), and second, through a perceptual decision task (13) to evaluate reward-motivated behavior. In particular, this experimental protocol suggests that anhedonia can be inferred from the response bias in an elementary discrimination task, where participants' performance can be asymmetrically reinforced. If our intuition is correct, we predict a diminished modulation of behavior by positive reinforcement (reward responsiveness) for introjective participants in comparison with the three other POE groups.

## Materials and methods

### Participants

One hundred seventy-seven undergraduate students participated in the study (mean age: 21.1, *SD*: 1.65, 106 women). As an important exclusion criterion, we did not select participants with self-reported symptoms of depression (mean BDI score: 9.11, *SD*: 6.8). Each participant completed one experimental session and received $7 USD. Informed consent forms were signed, and the guidelines of the Code of Ethics of the World Medical Association—Declaration of Helsinki were completely followed. The ethics committees of the Universidad del Desarrollo, Pontificia Universidad Católica de Chile, and the Universidad de La Frontera approved the study at all stages.

### Instruments and procedure

To determine the POE categories, the DEQ was administered. From the DEQ, two indexes were obtained: dependency and self-criticism scores. Through the interaction of these factors, four categories can be generated (normal, introjective, anaclitic, and mixed A-I). Additionally, each of the participants answered the BDI to confirm the presence of self-reported depression symptomatology. In addition, the BDI's anhedonia and melancholia subscales were used for analysis. These subscales were built according to Pizzagalli et al. (17) with the anhedonia subscale considering the sum of items associated with anhedonic symptoms—loss of pleasure (item 4), loss of interest (item 12), loss of energy (item 15), and loss of interest in sex (item 21)—and the melancholia subscale considering the sum of items associated with melancholic symptoms—loss of pleasure (item 4), guilty feelings (item 5), agitation (item 11), loss of interest (item 12), early morning awakening (item 16b), and loss of interest in sex (item 21). All questionnaires were randomly administrated 1 day before the experimental protocol.

**Figure 1:**
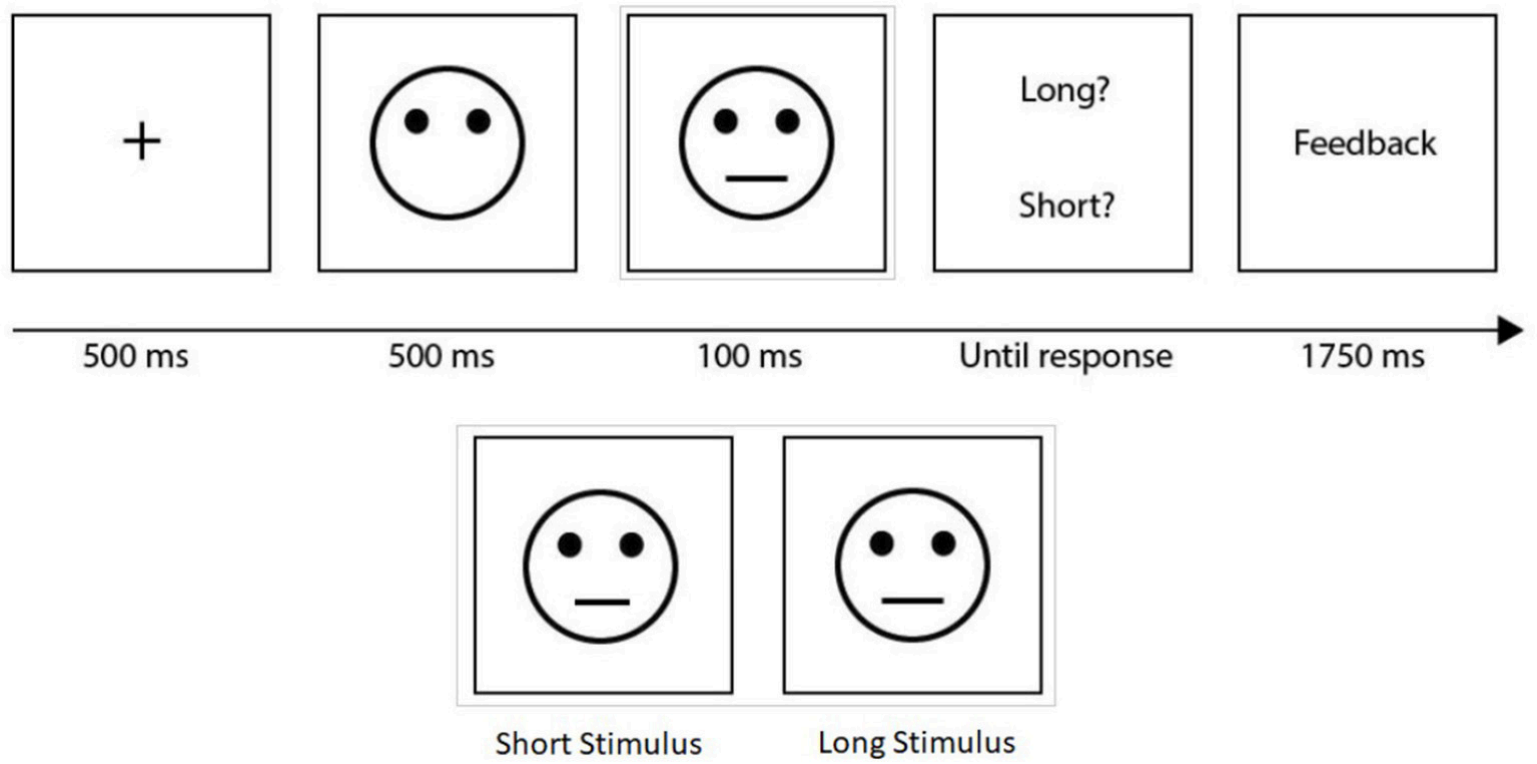
Perceptual discrimination task. After a fixation point, a mask-face without a mouth was presented. Immediately afterward, a second face with a long or short mouth was presented. Participants were asked to decide the length of the mouth (short/long). In some trials, a monetary reward was provided after the participant decision. Short and long stimuli were shown.

Regarding the experimental manipulation (Figure 1), participants performed a discrimination task (17, 18). After a fixation point (500 ms), a mask-face without a mouth was presented on the center of the screen (500 ms). Immediately afterward, a second face with a long (13 mm) or short (11.5 mm) mouth was presented (100 ms). Participants were asked to decide, in a separate response window, the length of the mouth (short/long), typing as fast as possible on a QWERTY keyboard (short: Q & long: W). In some trials, participants obtain a monetary reward after the decision. Participants were instructed that only a few correct answers would receive feedback. The task considers 300 trials divided into three equal blocks. Importantly, the task implemented a differential reinforcement program that objectively evaluates the tendency to change a response based on previous rewards. Thus, participants must choose between two options, only one being related to different probability of reward. One of the options is disproportionately reinforced (the rich stimulus, reinforced 30/40 of the trials per block) whereas the other is not (the lean stimulus, reinforced 10/40 of the trials per block). For half of the participants, correct responses on trials with the short mouth were the rich condition, and, for the other half of the participants, this was true for the long mouth. Given the probabilistic design, the participants could not infer which response was more advantageous based on the result of a single trial and, hence, had to consider the previous reinforcement to optimize their selections. The reinforcement procedure was carried out under a pseudo-random schedule in which it was determined which specific trial would be reinforced. If the participant did not correctly identify the stimulus in a trial intended to be reinforced, the feedback was delayed to the correct identification immediately afterwards.

### Analysis

Behavioral performance was analyzed using the signal-detection theory by calculating both response biases (Log-B) toward the more frequently rewarded stimulus (18, 19). Additionally, we investigate discriminability (Log-D) and performance (Response Times and Error Rate). Discriminability (Log-D) and Response bias (Log-B) were calculated considering the Pizzagalli et al. (18) formulae.

(1) $$\begin{array}{r} \text{Discriminability}:\log_{d}=\frac{1}{2}\log\left(\frac{Rich_{correct}*Lean_{correct}}{Rich_{incorrect}*Lean_{incorrect}}\right) \end{array}$$

(2) $$\begin{array}{r} \text{Response bias}:\log_{b}=\frac{1}{2}\log\left(\frac{Rich_{correct}*Lean_{incorrect}}{Rich_{incorrect}*Lean_{correct}}\right) \end{array}$$

In these two formulae, *Rich*_*correct*_ denotes the trials in which the participant responded correctly to the condition that had an enhanced reinforcement. On the other hand, *Lean*_*correct*_ considered the trials in which the participant responded correctly to the diminished reinforced condition. *Rich*_*incorrect*_ and *Lean*_*incorrect*_ refer to the trials in which the participant performed erroneously for each of the rewarded condition.

## Results

### Questionnaires

**Figure 2:**
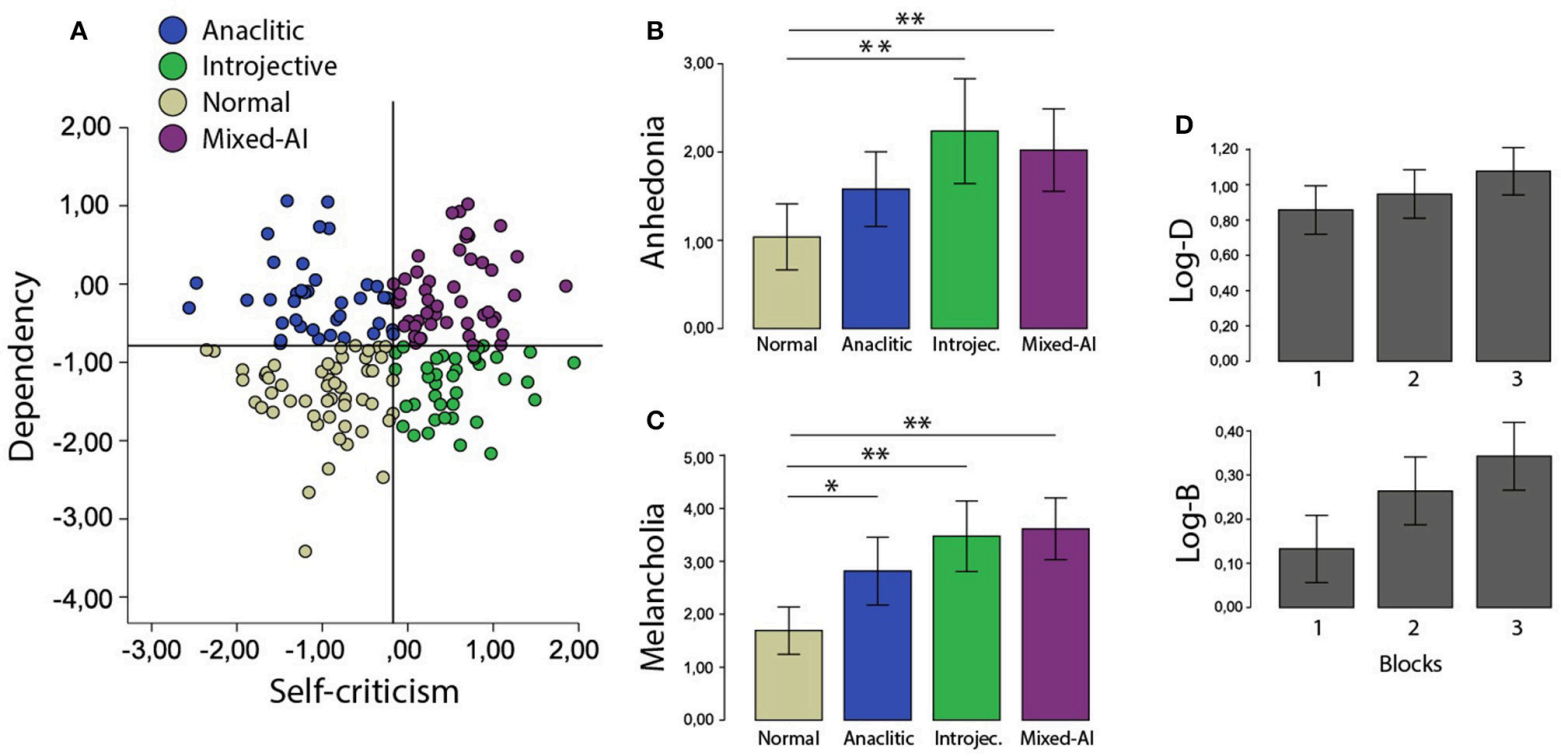
**(A)** Scatterplot to represent the cutoff points in the interaction between Dependency and Self-Criticism scores. Each point illustrates one participant and each color one category. **(B)** Melancholia and **(C)** Anhedonia for each POE group. **(D)** Discriminability and response bias along 3 Blocks. *P < 0.05 and **P < 0.01.

First, to determine the POE categories, the DEQ was analyzed by extracting Dependency and Self-criticism factors. The categorization limits used to determine the POE groups follow the standard literature criterion (15). The samples were similarly distributed across POE groups (normal: 52, introjective: 38, anaclitic: 38 and mixed AI: 49 participants, Figure 2A). The BDI questionnaire was employed to analyze anhedonia and melancholia. First, a one-way ANOVA on melancholia scores evidenced a significant main effect of group [*F*_(3, 176)_ = 10.51, *p* < 0.01, η2 = 0.15]. *Post-hoc* comparisons (Bonferroni) revealed that the normal group presented significant melancholia differences with all the other groups (normal vs. anaclitic ΔM = −1.12, *SE* = 0.40, *p* < 0.05; normal vs. introjective ΔM = −1.78, *SE* = 0.40, *p* < 0.01; normal vs. mixed A-I ΔM = −1.92, *SE* = 0.38, *p* < 0.01; Figure 2C). This result on melancholia suggests that there is a differential emotional pattern between the POE configuration and the normal characterization. Regarding anhedonia, a similar ANOVA evidenced a significant group effect [*F*_(3, 176)_ = 5.64, *p* < 0.01, η2 = 0.08, Figure 2B], which was characterized by significant differences between the normal and introjective groups and between the normal and mixed AI groups (normal vs. introjective ΔM = −1.20, *SE* = 0.32, *p* < 0.01, normal vs. mixed AI Δ*M* = – 0.98, *SE* = 0.30, *p* < 0.01 Bonferroni). This first result confirms our hypothesis, indicating that introjective individuals, our group of interest, differ from the normal characterization in terms of anhedonia. In the same line, the anaclitic group as introjective's counterpart does not differ in anhedonia from the normal group.

Finally, to rule out the possibility that our results could be interpreted simply as depression in the participants, i.e., a confounding effect of depression on anhedonia, we repeated the analysis considering six levels of depression (16). For this purpose, we ran a two-way ANOVA on the individual anhedonia averages with fixed Depression Level and POE Group factors. The analyses show a main effect of the Depression Level [*F*_(176, 3)_ = 42.85 *p* < 0.01]; however, no interaction is observed between this factor and the POE Group (*p* = 0.34), which allows us to rule out the possibility that the anhedonia variability reported above is simply explained by the level of depression in general.

### Experimental task

Results related to the experimental task show that there are no differences between POE groups' reward responsiveness and no diminished sensitivity to reinforcement in introjective individuals. Measurement of performance followed a similar pattern. First, Response Time (RT) and the mean Error Rate were analyzed using a repeated measures ANOVA with Blocks (1, 2, and 3) as within factors. POE groups (anaclitic, introjective, normal, and mixed AI) and Condition (rich and lean) were considered as between factors. We also included all possible interactions. Participants were considered the random factor. Before the analysis, mean RTs were log-transformed, and Error Rate was arcsine-transformed to approximate normal distribution. Regarding RTs, results indicated a significant main effect of Blocks [*F*_(1.9, 638.4)_ = 81.41, *p* < 0.01, η_*p*_^2^ = 0.19 Greenhouse-Geisser corrected] and Condition [*F*_(1, 342)_ = 5.03, *p* < 0.05, η_*p*_^2^ = 0.014] without any interaction among these three factors (all *ps* > 0.32) or with the POE main effect group (*p* > 0.17). Regarding performance, a significant main effect of POE group [*F*_(3, 344)_ = 3.38, *p* < 0.05, η_*p*_^2^ = 0.03] and an interaction between Blocks and Condition [*F*_(1.62, 558.1)_ = 4.33, *p* < 0.05, η_*p*_^2^ = 0.01, G-G] were observed. *Post-hoc* analysis (Bonferroni) showed significant differences between the anaclitic and normal groups (normal vs. anaclitic Δ*M* = 0.15, *SE* = 0.05 *p* < 0.05). No other effects were found (all *p*s > 0.37). In sum, results show that all groups improved their RTs through the task. Regarding the performance, only the anaclitic individuals showed POE group differences, improving their performance differentially through the Blocks of the task.

Second, the variables of interest were Discriminability (Log-D) and Response Bias (Log-B). The former denotes the subject's capacity to differentiate the two stimuli of the task (long or short mouth), and the latter represents the tendency to be impacted by the task reinforcement program. To test this, a repeated-measures ANOVA was run on Log-D and Log-B with factors of Blocks, POE group, and their interaction. Results for Discriminability showed a significant main effect of Blocks [*F*_(1.7, 303.9)_ = 13.1, *p* < 0.01, η_*p*_^2^ = 0.07, G-G corrected] with a difference between POE groups [*F*_(3, 171)_ = 2.69, *p* < 0.05, η_*p*_^2^ = 0.05] but without interaction between these factors (*p* = 0.75). In the same line, ANOVA on Response Bias again showed only a significant main effect of Block [*F*_(1.8, 313.3)_ = 12.7, *p* < 0.01, η_*p*_^2^ = 0.07]. No other effects were found (all *ps* > 0.66). Interestingly, through a linear regression, we could not show a relationship of this measure with the level of anhedonia (*ps* > 0.78) or with the level of depression (*ps* > 0.83). Consistent with the performance analysis (Error rate and RTs), Discrimination (Log-D), and Response Bias (Log-B) scores mainly increased as a function of Blocks. POE configurations were not significantly different in terms of reinforcement sensitivity (see Figure 2D).

Interestingly, the only important effect to highlight is that the anaclitic group significantly decreased its error rate in relation to the normal group. In other words, its performance increased among the blocks, and this was the POE group that performed best in the task. However, in relation to the results of Discriminability and Response Bias, this observation is not relevant in the context of our hypothesis.

## Discussion

The main objective was to investigate a psychological correlate of the POE configurations. In particular, we investigated whether a specific POE configuration (introjective individuals vs. others) presented a diminished motivation to reward or anhedonia. To this end, we classified the four POE configurations (normal, introjective, anaclitic, and mixed A-I). Then, we applied the BDI and evaluated sensitivity to reinforcement through a gold-standard experiment outlined in the anhedonia literature (18). It is important to mention that none of our participants presented a depression diagnosis; therefore, our results are unlikely to be biased on account of a psychopathological condition. Results showed that our prediction was only partially confirmed; while the questionnaire subscale denotes a differential anhedonic pattern in the introjective group, no differences were found between groups in the experimental task across the POE configuration. In sum, the experimental manipulation creates a bias with respect to how the individuals performed the task in all of the experimental groups, but no differences on discriminability and response bias between POE groups were observed.

Based on this result, it is not possible to characterize the response to reward according to POE configuration. While this may indicate that introjective individuals are not necessarily characterized by a lack of interest, some revisions to the application of the task in this context should also be considered. On one hand, this result could be explained by the response to reinforcement being just one aspect of anhedonia; it can also be experienced as the inability to feel pleasure or loss of interest and satisfaction in activities or even the lack of reactivity to usually pleasurable stimuli. On the other hand, this experimental task probably showed no differences associated with the POE configuration because these have been observed exclusively in depressive patients [i.e., (20)]. In this sense, the experimental task may not be as sensitive to detecting anhedonia trends in nonclinical populations.

However, the results related to the questionnaires did follow the direction of our hypothesis, having significant differences particularly for introjective individuals on BDI's anhedonia subscale. Moreover, if sensitivity to reinforcement was analyzed just between introjective and normal individuals, some incipient evidence appears to support this distinction. Undoubtedly, more and better evidence is needed to settle this question.

In conclusion, all these results could be considered preliminary evidence suggesting a potential difficulty experienced by introjective subjects in being stimulated by positive cues. The understanding of the POE has been wider than merely that of the perspective of psychopathology; however, the intensity of the depressive experience undoubtedly has clinical repercussions that manifest in mood and in its behavioral correlation. The anhedonia as part of the depressive constellation allows us to differentiate how the loss of interest is a symptom strained by one of the polarities of the experience.

## Author contributions

JS, CM, and MK conceived and designed the experiments. JS performed the experiments. JS and AV-C analyzed the data. JS, AV-C, MK, and CM wrote the paper.

### Conflict of interest statement

The authors declare that the research was conducted in the absence of any commercial or financial relationships that could be construed as a potential conflict of interest.

## References

[1] Blatt SJ. Levels of object representation in anaclitic and introjective depression. Psychoanal Stud Child (1974) 29:107–57. 10.1080/00797308.1974.11822616 4445397

[2] Blatt SJ Luyten P. A structural–developmental psychodynamic approach to psychopathology: two polarities of experience across the life span. Dev Psychopathol. (2009) 21:793–814. 10.1017/S0954579409000431 19583884

[3] Blatt SJ Quinlan DM Chevron ES McDonald C Zuroff D. Dependency and self-criticism: Psychological dimensions of depression. J Consult Clin Psychol. (1982) 50:113–24. 10.1037/0022-006X.50.1.113 7056904

[4] Luyten P Blatt SJ. Interpersonal relatedness and self-definition in normal and disrupted personality development: retrospect and prospect. Am Psychol. (2013) 68:172–83. 10.1037/a0032243 23586492

[5] Blatt SJ Shichman S Two primary configurations of psychopathology. Psychoanal Contemp Thought (1983) 6:187–254.

[6] Blatt SJ Polarities of Experience: Relatedness and Self-Definition in Personality Development, Psychopathology, and the Therapeutic Process. Washington, DC: American Psychologic (2008).

[7] Luyten P Sabbe B Blatt SJ Meganck S Jansen B De Grave C. Dependency and self-criticism: relationship with major depressive disorder, severity of depression and clinical presentation. Depress Anxiety (2007) 24:586–96. 10.1002/da.20272 17143851

[8] Fazaa N Page S. Dependency and self-criticism as predictors of suicidal behavior. Suicide Life Threat Behav. (2003) 33:172–85. 10.1521/suli.33.2.172.22777 12882418

[9] Reis S Grenyer BF. Pathways to anaclitic and introjective depression. Psychol Psychother. (2002) 75:445–59. 10.1348/147608302321151934 12626134

[10] Campos RC Besser A Morgado C Blatt SJ Self-criticism, dependency, and adolescents' externalising and internalising problems. Clin Psychol. (2014) 18:21–32. 10.1111/cp.12024

[11] Blatt SJ Experiences of Depression: Theoretical, Research and Clinical Perspectives. Washington, DC: American Psychological Association (2004).

[12] Hasler G Drevets WC Manji HK Charney DS. Discovering endophenotypes for major depression. Neuropsychopharma (2004) 29:1765–81. 10.1038/sj.npp.1300506 15213704

[13] Pizzagalli DA. Depression, stress, and anhedonia: toward a synthesis and integrated model. Annu Rev Clin Psychol. (2014) 10:393–423. 10.1146/annurev-clinpsy-050212-185606 24471371 PMC3972338

[14] Silva JR Vivanco-Carlevari A Barrientos M Martínez C Salazar LA Krause M. Biological stress reactivity as an index of the two polarities of the experience model. Psychoneuroendo (2017) 84:83–6. 10.1016/j.psyneuen.2017.06.016 28672276

[15] Blatt SJ D'Afflitti J Quinlan D. Experiences of depression in normal young adults. J Abnorm Psychol. (1976) 85:383–9. 10.1037/0021-843X.85.4.383 956505

[16] Beck AT Ward CH Mendelson M Mock J Erbaugh J. An inventory for measuring depression. Arch Gen Psychiatry (1961) 4:561–71. 10.1001/archpsyc.1961.01710120031004 13688369

[17] Pizzagalli DA Jahn AL O'Shea JP. Toward an objective characterization of an anhedonic phenotype: a Signal-detection approach. Biol Psychiatry (2005) 57:319–27r. 10.1016/j.biopsych.2004.11.026 15705346 PMC2447922

[18] Pizzagalli DA Iosifescu D Hallett LA Ratner KG Fava M. Reduced hedonic capacity in major depressive disorder: evidence from a probabilistic reward task. J Psychiatr Res. (2008) 43:76–87. 10.1016/j.jpsychires.2008.03.001 18433774 PMC2637997

[19] McCarthy DC Behavioral detection theory: Some implications for applied human research. In: Nevin JA Davison MC Commons ML editors. Signal Detection: Mechanisms, Models, and Applications. Hillsdale, NJ: Erlbaum (1991) p. 239–55.

[20] Vrieze E Claes SJ Anhedonia and increased stress sensitivity: two promising endophenotypes for major depression. Curr Psychiatry Rev. (2009) 5:143–52. 10.2174/157340009788971083

